# A Mechanistic Investigation on Cation-Modified Cellulose Nanofibrils–Reinforced Cement Composite

**DOI:** 10.3390/ma19010067

**Published:** 2025-12-23

**Authors:** Wei Tang, Tengfei Fu, Mingming Guo, Xixian Ji, Wendi Liu, Renhui Qiu, Demei Yu

**Affiliations:** 1 College of Transportation and Civil Engineering, Fujian Agriculture and Forestry University, Fuzhou 350108, China; 13107375955@163.com (W.T.); jixixian@cscec.com (X.J.); wendi.liu@fafu.edu.cn (W.L.); renhuiqiu@fafu.edu.cn (R.Q.); yudemei0826@fafu.edu.cn (D.Y.); 2 China West Construction Overseas (Chengdu) Co., Ltd., Chengdu 610200, China; guo_mingming@cscec.com; 3 CSCEC Strait Construction and Development Co., Ltd., Fuzhou 350015, China

**Keywords:** cellulose nanofibril, cement, cation modification, mechanical properties, shrinkage and cracking, microstructure analysis

## Abstract

Cellulose nanofibril (CNF), as a renewable biomass material, has the characteristics of low density, high strength, and high hydrophilicity. It can also overcome shortcomings of traditional inorganic nano materials, such as difficult dispersion, high cost, and high health risks. In this work, CNF was modified with a cationic surfactant to further enhance the compatibility with hydrating cement. The effects on cement paste were assessed via compressive and flexural strength, heat of hydration, and restrained ring cracking. The reinforcing mechanisms were analyzed by microhardness test, XRD, and BSE-SEM/EDS. Results showed that cation-modified CNF improved mechanical performance, with an optimal dosage of 0.15 wt.% (by binder). Restrained ring test showed that cation-modified CNF–cement composite delayed crack initiation. An isothermal calorimetry test revealed that cation-modified CNF can increase hydration rate in early age. Microstructural analysis confirmed promotion of denser hydration products. A comprehensive consideration of experimental results indicates internal curing and “short-circuit diffusion” are likely the enhancing mechanism.

## 1. Introduction

Cementitious materials are the most widely used man-made materials in civil engineering worldwide, and it was reported that the cement and concrete industry accounts for 5–7% of global CO_2_ emissions [1]. Additionally, traditional cementitious materials suffer from low toughness and inadequate flexural and tensile strength, failing to meet the high-performance demands required by complex external environments and specialized applications [2]. Thus, the long-term development of cementitious materials necessitates innovation, and the introduction of new, high-performance green cementitious materials is a crucial industry challenge.

To reduce energy consumption and enhance the properties of cementitious materials, industrial by-products such as fly ash, slag, and silica fume are extensively utilized as cement replacement materials. Admixtures are also employed to improve the workability of concrete [3]. With the development of nanotechnology in recent years, nanomaterials have been increasingly incorporated into cementitious materials to improve their performance [4]. Nanomaterials exhibit unique effects, such as filler effects [5], pozzolanic effects [6], nucleation effects [7,8,9], and micro-bridging effects [10]. These effects facilitate cement hydration, reduce porosity, enhance mechanical properties, mitigate shrinkage, and improve impermeability and durability. For instance, inorganic nanomaterials like nano-silica can improve the water absorption properties of cementitious materials, densify the microstructure, and enhance the compressive strength of the cement paste [11]. Despite these benefits, inorganic nanomaterials are often costly and not cost-effective, and some may pose health risks [5].

Nanocellulose, a promising biomass nanomaterial, offers excellent properties including biodegradability, biocompatibility, and environmental friendliness. Compared to inorganic nanomaterials, nanocellulose is abundant, cost-effective, readily available, and can be easily modified due to its high hydroxyl content, making it a material with broad application prospects [12].

Plants are the primary source of nanocellulose fibers, which can be produced through methods such as mechanical, acid hydrolysis, and biological processes [13]. Treated nanocellulose can be categorized by form into cellulose microfibrils (CMFs) [14,15], cellulose nanofibers (CNFs) [16,17,18,19,20,21], and cellulose nanocrystals (CNCs) [22,23,24,25,26,27]. CNFs have diameters ranging from 4 to 20 nm and lengths from tens to hundreds of nanometers [28]. They are characterized by a high elastic modulus (100–140 GPa), high tensile strength (7500 MPa), high aspect ratio (70), large specific surface area (150–250 m^2^/g), relatively low density (1.6 g/cm^3^), and high reactivity [29]. In recent years, many works have been performed using CNF with cementitious materials to improve strength [15,16,20,21], stiffness [16], toughness [15,19], and reduce porosities [20] with green and low-carbon requirements, expanding the application domains of cementitious materials. However, the dispersion and interfacial bonding of CNFs with cement matrices can be challenging due to their hydrophilic nature (fighting with cement particles for water molecules) and tendency to agglomerate (near zero zeta potential), which may limit their effectiveness in reinforcing the composite. In a previous study, a CNF made from bamboo pulp was modified with 3-mercaptopropyl-trimethoxysilane (KH590) to be used with autoclaved aerated concrete (AAC) [30]. This study confirmed that the siloxane groups of KH590 interact with the hydroxyl groups of cellulose nanofibrils (CNFs), resulting in the grafting of hydrophobic moieties onto the CNFs and enhancing their interfacial bonding with the AAC matrix.

Inspired by these findings, to overcome the compatibility and dispersion challenge, cation-modified cellulose nanofibrils have the potential to address the inherent challenges in dispersing CNFs in cement, which include their natural hydrophilicity and tendency to agglomerate. It was hypothesized that by using a cationic surfactant, due to the fact that most calcium silicate phases in OPC are negatively charged, the compatibility of CNFs with the cement matrix and the performance of cation-modified CNF–cement composite can be further improved. The mechanism of enhanced performance is underexplored and can provide insights into offering a promising alternative to traditional inorganic nanomaterials in cementitious materials.

In this study the effects of nanocellulose fibers were investigated on the mechanical properties, microstructure, hydration heat, and shrinkage cracking of cementitious materials. Based on these experimental results, hypotheses of enhancing mechanisms were carefully examined.

## 2. Materials and Methods

### 2.1. Cation-Modified CNF

The original CNF was produced from enzymatic hydrolysis of cellulose fiber board (>96% purity) in the laboratory facility in Qishan Campus in Fujian Agriculture and Forestry University. A piece of 50 g cellulose fiber board was thoroughly shredded by a mechanical pulverizer then mixed with 1250 mL deionized water and 4 g of cellulase in a reaction vessel. Then, a sodium citrate buffer solution was added to the vessel to adjust the pH to 5.0 at 60 °C. Under mechanical stirring and ultrasonic dispersion, the enzymatic hydrolysis was continued for 10 h. Then, the suspension was placed in a beaker and heated in a water bath to 100 °C for 10 min to inactivate the enzyme. After cooling to room temperature, CNF from the suspension was separated by suction filtration and washing, washed thoroughly three times with deionized water, then placed in a dialysis bag for 48 h. Then the final product of CNF (approximately 30 g in a suspension state) was collected for future modification.

The etherification reaction of cation-modified CNF was conducted in a 2000 mL beaker. A water bath was preheated to 60 °C, after which 100 g (dry weight) of original CNF, 75 mL of 0.5 mol/L sodium hydroxide, and 200 mL of isopropanol were mixed and placed into the beaker. The beaker was sealed with plastic wrap (to prevent excessive evaporation) and heated in the water bath for 60 min. Subsequently, 125 g of 2,3-Epoxypropyltrimethylammonium chloride (EPTMAC) added at a ratio of 1.25 g/g of CNF) and 2 L of isopropanol were added to the beaker. The mixture was stirred every hour for 5 min over a total reaction time of 10 h. After completing the cationic modification, samples were washed with deionized water until a neutral pH was reached. The suspension was homogenized using a high-pressure homogenizer (AH100D, ATS, Cambridge, ON, Canada) at 100 MPa for eight cycles to produce the cation-modified CNF suspension. This modification was following procedures described in the literature [31], with slight alteration.

**Figure 1:**
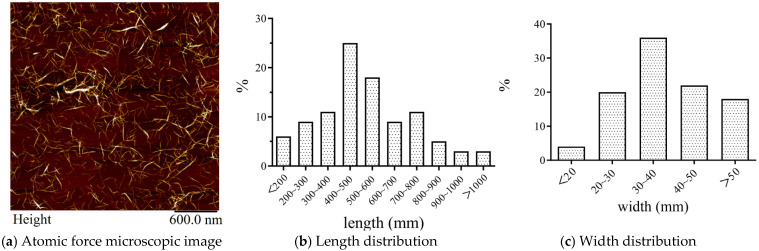
Fiber dimensions of cation-modified CNF.

The resulting cation-modified CNF suspension has a solid concentration of 1.2 wt.%. The length distribution of cation modified CNF, observed by a 5500AFM (Agilent, Santa Clara, CA, USA), is shown in Figure 1a. A distribution of length and width of CNF is shown in Figure 1b,c. The average aspect ratio (length to width) is 21.

**Figure 2:**
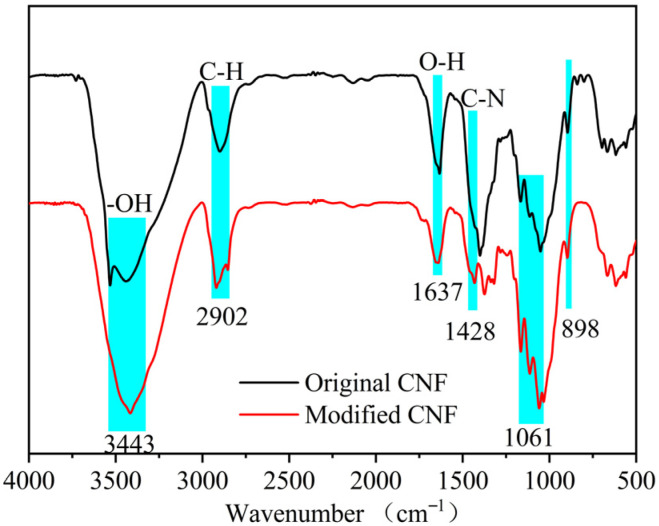
Infrared spectrum of CNF.

Figure 2 presents the infrared spectrum (Vertex 70, Bruker, Ettlingen, Germany) of cation-modified CNF, featuring functional groups at 3443 cm^−1^, 2902 cm^−1^, 1637 cm^−1^, 1162 cm^−1^, and 898 cm^−1^. The absorption peak at 1428 cm^−1^ corresponds to the C-N stretching vibration, indicating that the hydroxyl groups in the nanocellulose fibers were substituted by quaternary ammonium groups, confirming successful fiber modification. The zeta potential was measured to be +54.6 mV.

**Figure 3:**
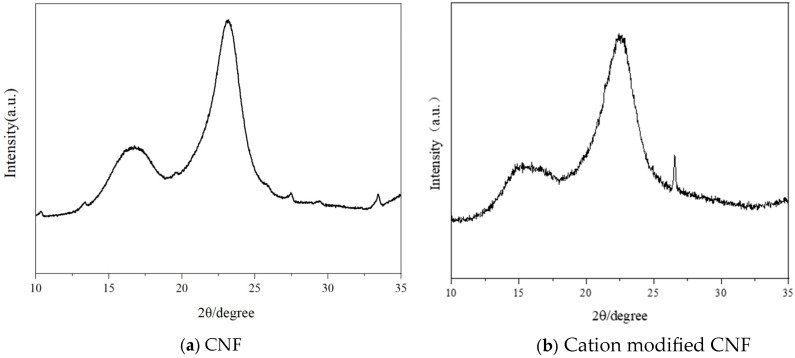
XRD of (**a**) original CNF and (**b**) cation-modified CNF.

The X-ray diffraction of cation-modified CNF in Figure 3 reveals a primary crystalline peak at 22.5° for the (002) plane, with peaks at 15° and 16° for the (001) plane, and a sharp intense peak at 26.5° for the (004) plane. The crystallinity of unmodified CNF and cation-modified CNF is calculated to be 69.4% and 73.6%, respectively, representing a typical Cellulose I structure. The high crystallinity imparts good stability and a high elastic modulus, which is in favor when using with cement.

### 2.2. Preparation of CNF–Cement Composite Samples

In this study, cation-modified CNF was mixed with ordinary Portland cement at varying dosages (0 wt.%, 0.1 wt.%, 0.15 wt.%, 0.2 wt.%, 0.25 wt.%) to prepare a slurry with a water-to-cement ratio of 0.3. A local PO 42.5 cement was used, and the composition is shown in Table 1.

The CNF–cement composite preparation process was as follows:

- The weighed CNF was placed in water and dispersed using a magnetic stirrer for 5 min to obtain a CNF suspension.
- The weighed cement was placed in a vacuum mixer (Twister Evolution, Renfert, St. Charles, IL, USA), and the CNF solution was added. The mixture was stirred under vacuum (at 200 rpm) for 2 min.
- The mixer was then set to 300 rpm for an additional 1 min to obtain the CNF cement paste.
- The cement paste from the vacuum mixer was stirred evenly with a small spatula for 30 s.
- Once the CNF cement paste was prepared, it was poured into standard molds coated with a release agent in two batches. During each pouring, a spatula was continually used to insert and compact the mixture. After filling, the molds were placed on a vibration table for 30 s to reduce air bubbles in the paste with excess paste scraped off the sides.
- The specimens were labeled and covered with plastic wrap for indoor curing for 24 h. After demolding, they were placed in a humidity chamber with RH greater than 95% until the specified curing ages (3, 7, and 28 days).

### 2.3. Experimental Methods

Mechanical properties were measured using an electronic universal testing machine (Instron, Norwood, MA, USA) with specimen dimensions of 20 mm × 20 mm × 20 mm for compressive strength tests and 20 mm × 20 mm × 80 mm for flexural strength tests, at 3, 7, and 28 days. The testing procedure followed China Standard GB/T 17671-2020 [32].

The hydration heat rate and cumulative heat release of the CNF–cement composites were measured using an isothermal calorimeter (TAM Air, TA Instrument, New Castle, DE, USA) over 7 days to analyze the effect of CNF on cement hydration heat. Prior to the experiment, ensure stable room temperature and weigh all materials.

For the XRD analysis (Ultima IV, Rigaku, Tokyo, Japan) of hydration products, samples from both the control group (0 wt.% CNF) and the optimal dosage group (0.25 wt.% CNF) were tested after 28 days of hydration. Appropriate amounts of samples were mixed with isopropanol and ground before being dried in a vacuum desiccator and passed through a 200-mesh sieve. The scanning range of 10–80° and a scanning speed of 10°/min.

Backscattered electron microscopy (BSEM) was used to identify phase distribution by Verios G4 SEM (Thermo Scientific, Waltham, MA, USA). Energy-dispersive spectroscopy (EDS) was used to quantitatively reveal elemental composition of different phases. Samples hydrated for 28 days with 0.25 wt.% CNF were first immersed in isopropanol for 2 days to replace the free water in the samples. The samples were then placed in a vacuum desiccator to dry. After drying, the samples were cut into small pieces then carefully ground and polished within one hour, then sealed with silica gel beads desiccant before testing to minimize carbonation.

**Figure 4:**
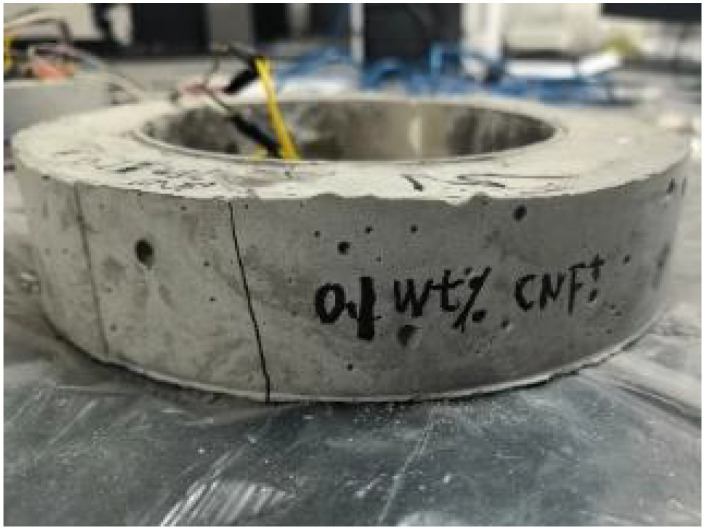
Image of restrained ring test.

The cracking resistance of cement was evaluated using a restrained mini ring test, which was adopted from ASTM C1581 [33]. The rings used had an outer diameter of 90 mm, an inner diameter of 60 mm, and a height of 40 mm (as shown in Figure 4), with CNF dosages of 0.05 wt.% and 0.1 wt.%. It should be noted that to better capture the potential influence of autogenous shrinkage and internal curing effect, a lower water-to-cement ratio of 0.3 was used.

The specific procedures were as follows:

- The base plate and the inner ring, onto which strain gauges had been affixed, were thoroughly cleaned. The inner and outer rings were securely fixed before pouring the cement paste into the mold. The paste was placed in two layers, and each layer was vibrated to ensure good compatibility. After pouring, the surface of the specimen was leveled and covered with plastic wrap.
- The specimens were cured indoors for 24 h before removing the outer ring. The specimens were covered with plastic wrap to prevent moisture loss.
- The strain gauges were connected to a data acquisition system for monitoring, with readings taken every 10 min for a testing duration of 14 days.

**Figure 5:**
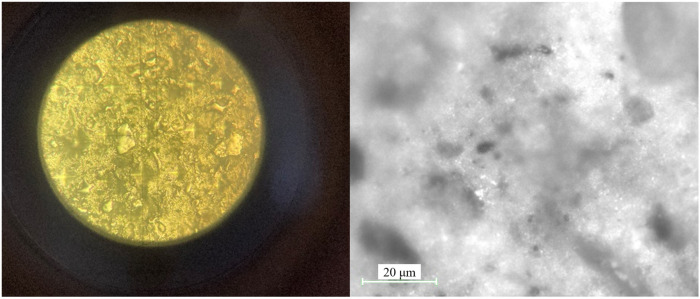
Images of microhardness testing.

The preparation method for the microhardness testing samples followed the same procedure as that for the BSEM samples. After preparation, the surfaces of the samples were gold-coated to enhance conductivity, facilitating the observation of indentation width. The HVS-1000 digital microhardness tester was used to load the specimens that had been cured for 7 days, applying a load of 0.025 N and maintaining it for 5 s before unloading. Figure 5 shows images taken from the microhardness test.

## 3. Results and Discussions

### 3.1. Mechanical Properties

**Figure 6:**
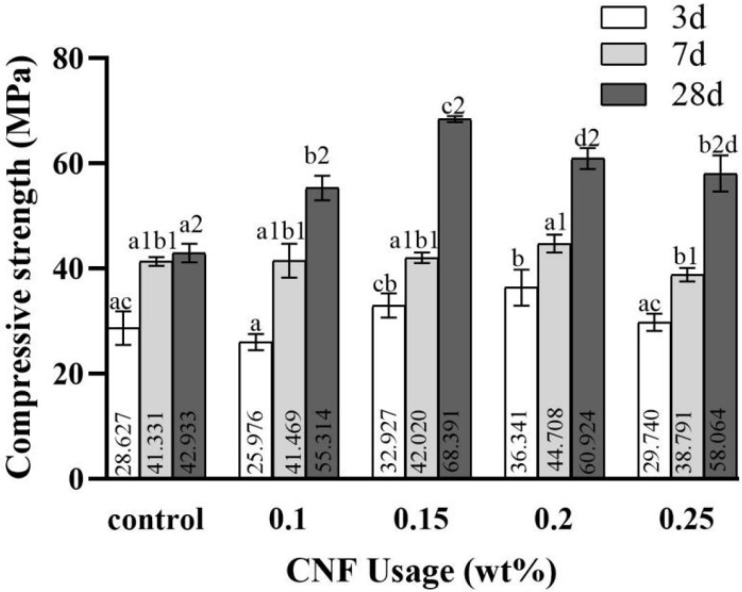
Compressive strength. Data were analyzed with one-way ANOVA. Significant differences exist between any two groups when they do not share a common designation over the columns.

Figure 6 illustrates the impact of varying dosages of cation-modified CNF on the compressive strength of cement at three curing ages. The results indicate that the addition of cation-modified CNF positively affects compressive strength across different ages, overall demonstrating an initial increase followed by a decline. After standard curing for 3 days, the compressive strength increased for all dosages except for 0.1 wt.%, which showed a slight decrease. Notably, a dosage of 0.2 wt.% yielded the most substantial improvement at 26.95%. By 7 days, there was no significant enhancement in compressive strength, with the optimal dosage showing an increase of only 8.17%. However, by 28 days, the strength increase was most pronounced, particularly at the 0.15 wt.% dosage, which resulted in a 59.30% improvement. These findings suggest that the optimal dosage of cation-modified CNF enhances the compressive strength of cement. The matrix strength of the cement paste depends on hydration products and microstructure. Cationic CNF has water-retaining properties, promoting the continued hydration of unhydrated cement particles and increasing hydration products, thereby enhancing compressive strength. However, the strength trend of the specimens exhibited an increase followed by a decrease, likely due to CNF agglomeration leading to stress concentration. This founding is consistent with previous work [19].

**Figure 7:**
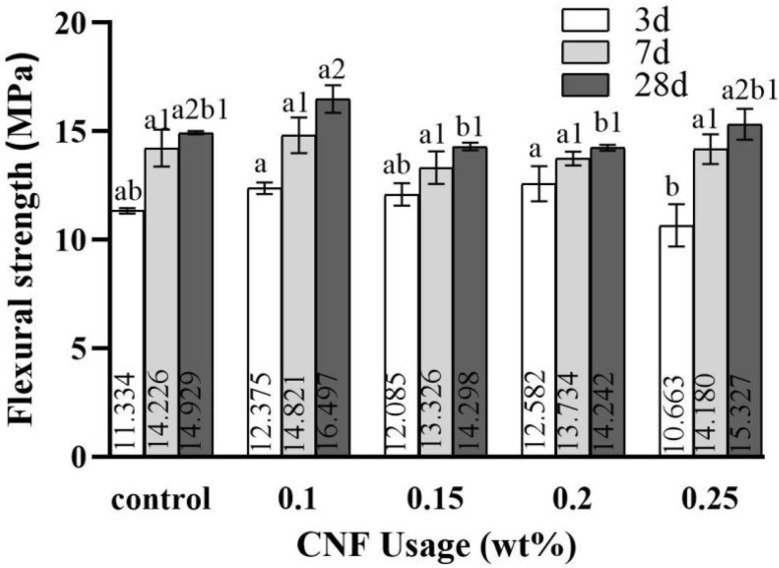
Flexural strength. Data were analyzed with one-way ANOVA. Significant differences exist between any two groups when they do not share a common designation over the columns.

Figure 7 displays the flexural strength results. At the optimal dosage, the strengths of the cement specimens at 3 days, 7 days, and 28 days increased by 11.0%, 4.33%, and 10.5%, respectively. In comparison to compressive strength results, flexural strength did not exhibit clear trends. Among the different dosages, 0.1 wt.% CNF led to the most significant enhancement in flexural strength.

### 3.2. Heat of Hydration

#### 3.2.1. Regular Samples with No Extra Water on Top Surface

**Figure 8:**
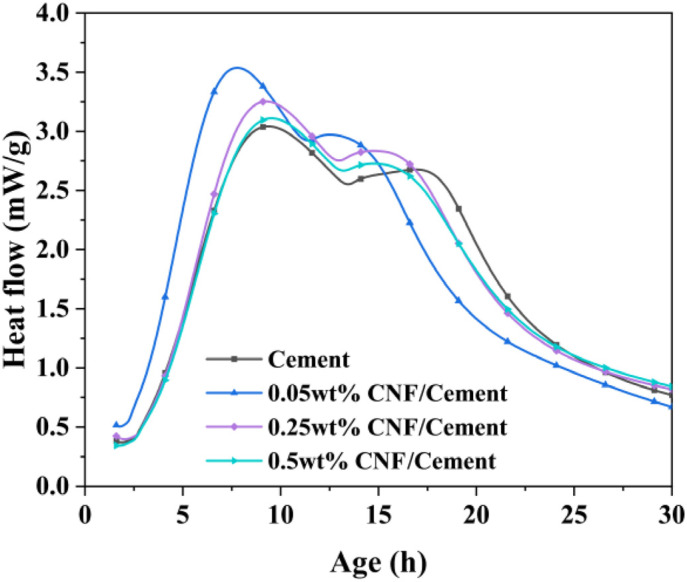
Hydration heat release rate for the first 30 h.

**Figure 9:**
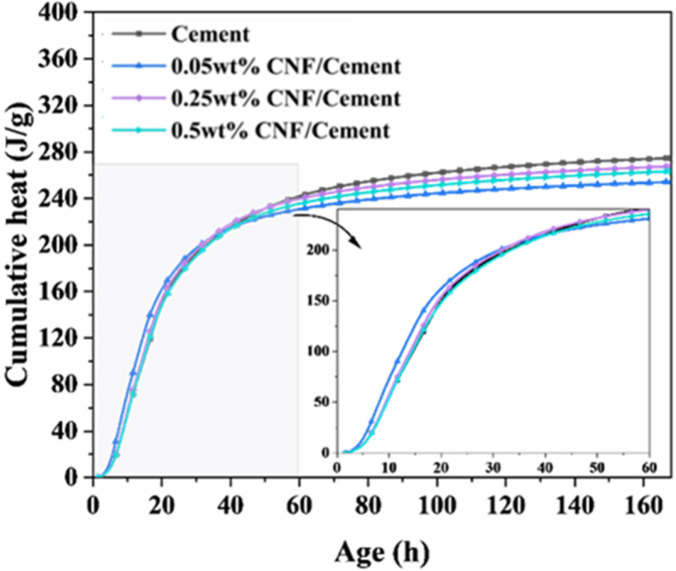
Cumulative heat at 7 days.

The hydration rate and cumulative heat of ordinary Portland cement containing cation-modified CNF at various dosages are illustrated in Figure 8 and Figure 9. These figures reflect changes in hydration rates and cumulative heat release for each mixture, with the corresponding cumulative heat at 7 days provided in Table 2.

The incorporation of cation-modified CNF facilitates early hydration heat release of the cement paste, accelerating the initial reaction rate and elevating peak heat release values. Lower doses of CNF showed more pronounced effects on early hydration heat release, which aligns with mechanical performance results. Cationic CNF binds closely to the surface of cement particles and accelerates early reaction rates. However, excessive CNF may compete for surface moisture, leading to reduced reaction rates at higher dosages. During the first 40 h, the cumulative heat of the CNF-incorporated paste exceeded that of the control group, but diminished thereafter, indicating that CNF’s effects on cement primarily concentrate in the early stages.

#### 3.2.2. Water-Sealed Samples

To investigate the potential working mechanism of cation-modified CNF, a mall batch of heat of hydration samples were water sealed by adding approximately 3 g of water on top of each sample. The water-sealed conditions’ impact on hydration heat of the cement is summarized in Table 3. It can be seen that by adding extra water, the surface heat of hydration of all mixtures was increased. The mixture with 0.05 wt.% CNF showed the most significant increase (10.2%), almost twice as much as the control cement mixture (5.20%). This result may be attributed to the “micro-tunnels” formed inside cement paste further facilitating moisture transport. This phenomenon was first depicted in the literature [22] referred as “short circuit diffusion” for CNC-cement composite. As the CNF dosage increased, this effect was diminishing likely because the amount of CNF was surpassing the percolation threshold, where the agglomeration would start to negate the “short circuit diffusion” effect. This phenomenon was also discovered with the CNC–cement composite [23].

### 3.3. Restrained Shrinkage

The ring restraint apparatus utilizes the constraining effect of an internal steel ring to inhibit shrinkage deformation in cement specimens. Under the action of the inner rigid ring, the internal net paste ring is subjected to tensile stress [34]. By collecting data from strain gauges, the precise stress values at that moment can be accurately determined. When the net paste reaches its ultimate tensile strain, cracking occurs, which appears as a sudden change toward neutral in strain data.

**Figure 10:**
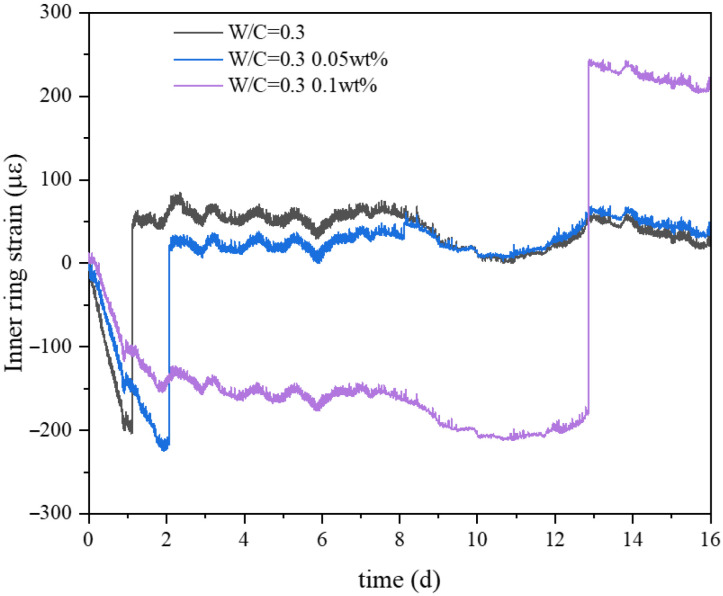
Strain in the restrained rings.

Following the ASTM C1581 [33], potential risks of ring-shaped cracking in concrete were evaluated. Data collected from strain gauges over 16 days shows the relationship between the internal rigid ring strain and time as shown in Figure 10. It can be observed that the control group experienced cracking on the first day, while the cracking times for the 0.05 wt.% CNF and 0.1 wt.% CNF groups were on the second and thirteenth days, respectively.

**Figure 11:**
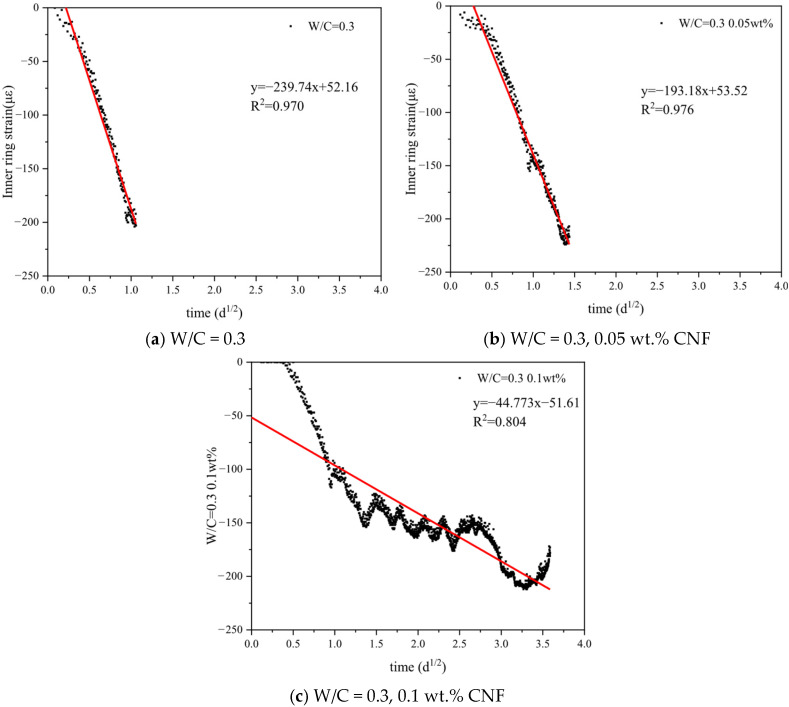
Strain rate calculation in the restrained rings.

The linear relationship between the strain of concrete’s internal rigid ring and the square root of time was determined using a fitting function to calculate the strain rate factor for each group, with results shown in Figure 11.

Based on results in Figure 10 and Figure 11, the relevant parameters were obtained and presented in Table 4. The presence of CNF resulted in a decrease in strain rates of 19.4% and 81.3% for the 0.05 wt.% and 0.1 wt.% CNF dosages, respectively. The incorporation of CNF delayed the cracking time of the cement composites and reduced the risk of cracking, indicating an enhancement in the cracking resistance of the cement paste due to the addition of CNF.

### 3.4. XRD Analysis of Hydrated CNF–Cement Composites

**Figure 12:**
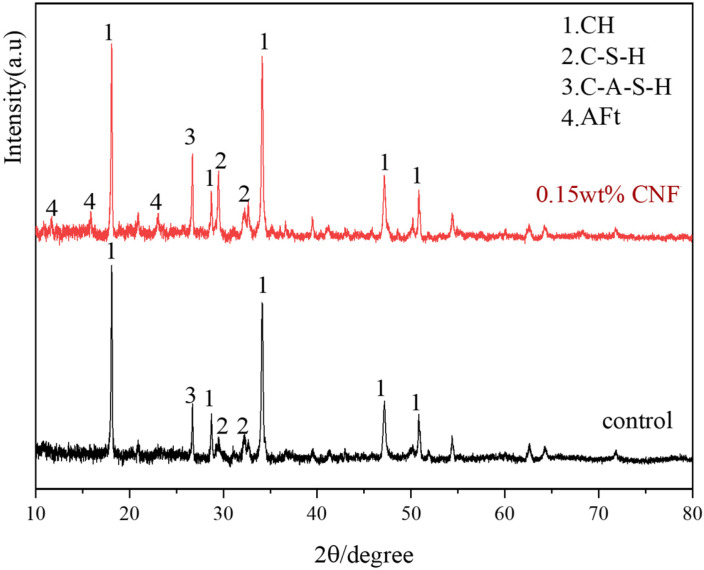
XRD analysis of CNF–cement composite.

Figure 12 demonstrates the influence of cation-modified CNF on the hydration products of cement. Under standard curing conditions for 28 days, it is evident that CNF promotes the generation of more hydration products. The XRD peaks for calcium hydroxide at diffraction angles of 18° and 34° (representing Ca(OH)_2_) were higher than those of the control group, indicating higher degree of hydration. The peaks at 11.6° and 15.9° correspond to ettringite, suggesting that the addition of cation-modified CNF significantly enhances the formation of this hydration product. Peaks at 29.5° and 32° are associated with C-S-H gel, while the peak at 26.7° corresponds to C-A-S-H gel. The observed increases in the C-S-H and C-A-S-H hydration products indicate improved mechanical performance relative to the control group, demonstrating that the addition of CNF increases the hydration degree of cement.

### 3.5. Microhardness

**Figure 13:**
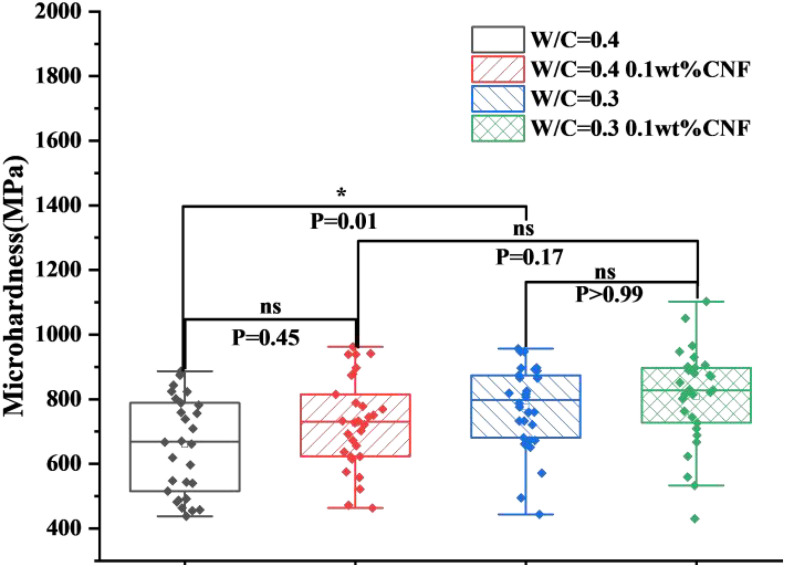
Statistical presentation of microhardness results of CNF–cement composites of 0.3 and 0.4 *w*/*c*.

Figure 13 displays the microhardness testing results of cement composite specimens with and without CNF incorporation at 7 days with different *w*/*c*. In general, both the CNF and non-CNF mixtures with 0.3 *w*/*c* exhibited significantly higher hardness values compared to 0.4 *w*/*c* mixtures, indicating that more high-density C-S-H were formed at the lower *w*/*c*. The addition of CNF further increased the hardness values. For 0.4 *w*/*c*, the median hardness was 669.63 MPa, while the inclusion of 0.1 wt.% CNF increased the median to 727.85 MPa, representing an increase of 8.69%. Specifically, at a 0.3 water-to-cement ratio, the median hardness was 797.72 MPa, compared to 826.83 MPa with the addition of 0.1 wt.% CNF, indicating an increase of 3.65%. The data for the CNF-incorporated group were more concentrated with less scatter, which was consistent with previous work [35]. The addition of CNF can effectively enhance hydration and form more high-density C-S-H, which aligns with the findings from the mechanical performance and hydration heat results.

### 3.6. Microstructure Analysis Based on SEM/EDS

**Figure 14:**
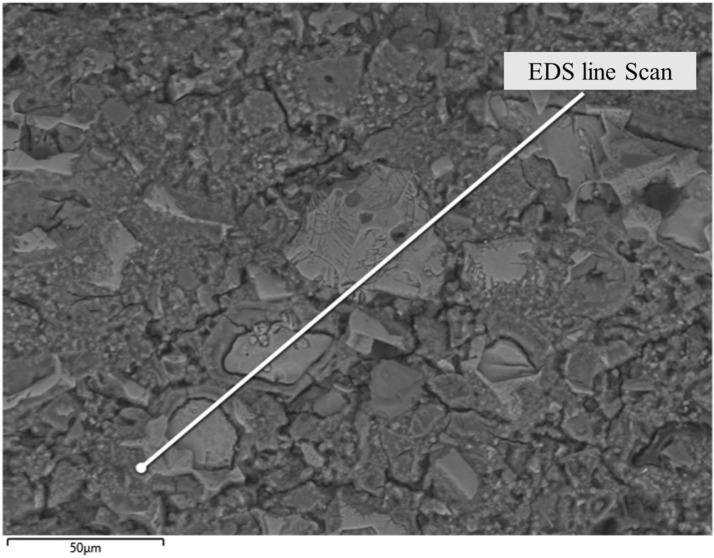
SEM image of CNF–cement composite (solid line indicating the EDS line scan location).

Backscattered electron microscopy (BSEM) images of cement allow for phase confirmation based on grayscale and morphological characteristics. Materials with higher density and average atomic number exhibit greater brightness. In BSEM images, the brightness order from bright to dark is as follows: unhydrated cement particles, calcium hydroxide (CH), C-S-H gel, and porosity or cracks. C-S-H gel can be categorized into internal and external hydration products based on grayscale; internal hydration products are formed at the boundary of cement particles and exhibit a denser structure and brighter grayscale. In contrast, external hydration products fill capillary pores and exhibit a more porous structure, appearing darker. Figure 14 shows a BSE image of CNF–cement composite.

**Figure 15:**
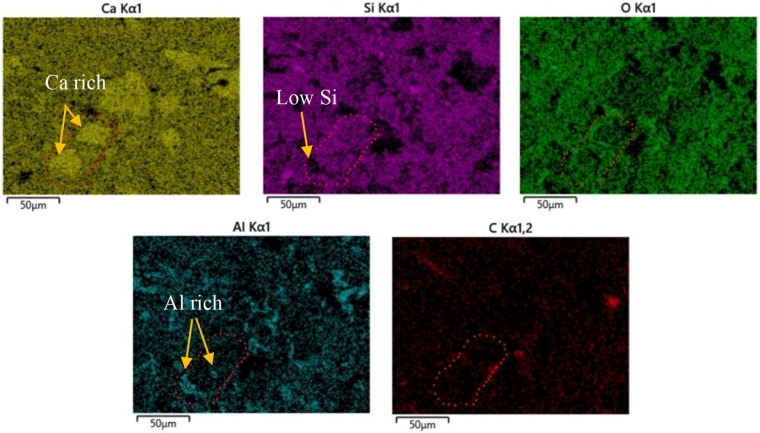
EDS elemental mappings of CNF–cement composite.

Energy dispersive spectroscopy (EDS) allows for elemental qualitative analysis of micro-regions. Figure 15 presents a backscattered image of localized hydration products in CNF–cement composite, where EDS data reveal high calcium (Ca) content in the marked regions, which appear prominently highlighted, while silicon (Si) content is relatively lower at the forefront. The content of oxygen (O) and carbon (C) appears uniform, with elevated aluminum (Al) content indicating these are aluminate phases.

**Figure 16:**
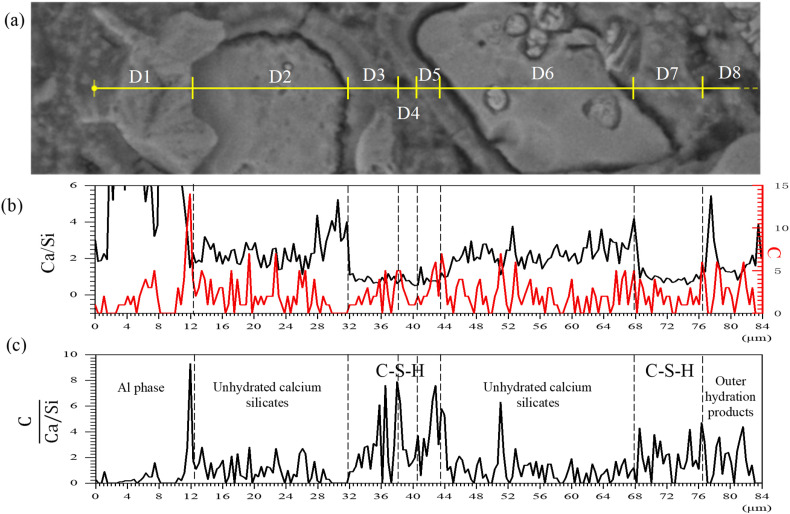
(**a**) A selected “line” region; (**b**) Ca/Si value (to the left) and carbon (C) intensity (to the right); (**c**) C/(Ca/Si) value in different regions along the “line”.

With all the microscopic information considered, a better perspective at looking at the enhancing mechanism of CNF is presented in Figure 16. Figure 16a illustrates a selected “line” region under observation, wherein the marked areas are categorized into eight regions based on morphological and brightness characteristics. D1 is likely an Al-rich phase such as AFt. D2 and D6 are hydrating cement particles (likely C_3_S phase) based on Ca/Si. D3, D4, D5, D7, and D8 are hydrated phases. The most interesting regions are D3, D4, and D5 due to the fact that this small region demonstrates a typical “ring” structure during hydration. To be specific, D3 and D5 are the inner hydration products which contain high density C-S-H, which evidently has the lowest Ca/Si in Figure 16b, forming during the cement particle hydrating toward the inner region. D4 and D7 are like the outer hydration product (low density C-S-H growing outward). Carbon concentration shown in Figure 16b indicates the concentration of CNF. It should be noted that boundaries between particles (positions at 12, 38, 44, 68, 76 μm) are rich in carbon (CNF), which is also consistent with previous work [23]. This is because the cation-modified CNF are more prone to be attached to silicate phases (negative zeta potential) due to its positive zeta potential. Therefore, the value of C/(Ca/Si) demonstrated in Figure 16c reflects the influence of the CNF-rich regions on the interface. A higher C/(Ca/Si) suggests that more high-density hydration products are formed under the influence of CNF.

### 3.7. Additional Discussions on Potential Mechanisms

In general, there are five potential working mechanisms of cation-modified CNF such as seeding effect, mechanical reinforcement, crack bridge effect, internal curing effect, and “short circuit diffusion”. Taking all experimental results into comprehensive consideration, a summary of potential working mechanisms is given in Table 5. It is noted that the plus sign (“+”) means the evidence works in favor of the corresponding hypothesis, and minus sign (“−“) means against.

In sum, the mostly likely enhancing mechanism of cation modified CNF are internal curing and “short circuit diffusion”, which is consistent with previous research [16,20,21,22,23,24,25].

## 4. Conclusions

This study presents a mechanistic investigation on cation-modified CNF–reinforced cement composite. It explores the influence of different CNF dosages on the mechanical properties of cementitious materials, examines the impact of CNF on hydration heat release using isothermal calorimetry test, and assesses cracking resistance through restrained ring tests. XRD was utilized for characterizing hydration products and chemical composition, while BSE/EDS Mapping was employed to analyze the microscopic morphology of the products, thus determining the enhancing mechanisms of cation-modified CNF are likely internal curing effect and “short-circuit diffusion”. The main conclusions are as follows:

- With increasing CNF content, the strength initially increases and then decreases, suggesting that an optimal dosage of 0.15 wt.% CNF can enhance the mechanical performance of cement.
- Restrained ring test (with sealed specimens) results indicates that CNF effectively reduces autogenous shrinkage and delays crack formation, likely due to internal curing effects.
- Hydration Heat Analysis: The addition of CNF increases the rate of hydration heat release in cementitious materials, thereby enhancing the generation of hydration products. Water sealing increases its cumulative heat release, suggesting that CNF can enhance the reaction rate of cement under sufficient moisture conditions, supporting the short-circuit diffusion hypothesis.
- XRD tests show that CNF increases the generation of hydration products. BSE/EDS mapping reveals that CNF is mainly distributed in the shell region, supplying moisture for cement particle hydration, improving the microscopic morphology and promoting high-density hydration product, thus validating the short-circuit diffusion hypothesis.

## Figures and Tables

**Table 1:** Cement chemical composition.

Component	CaO	SiO_2_	Al_2_O_3_	Fe_2_O_3_	SO_3_	MgO	Others
Mass (%)	59.30	21.28	5.99	3.31	2.51	2.16	5.54

**Table 2:** Heat of hydration of different CNF dosages (cumulative heat at 7 days).

Mixture	Cumulative Heat (J/g) at 7 Day	Relative Change (Compared to Neat Paste)
Neat paste	289.94	0
0.05 wt.% CNF	267.56	−8.03%
0.25 wt.% CNF	279.31	−2.69%
0.5 wt.% CNF	274.24	−4.27%

**Table 3:** Heat of hydration (cumulative heat at 7 days).

Mixture	Cumulative Heat (J/g) at 7 Day	Relative Change (Without/with Water Seal)
Neat paste	274.76	+5.20%
Neat paste (water seal)	289.94	
0.05 wt.% CNF	254.34	+10.2%
0.05 wt.% CNF (water seal)	283.22	
0.25 wt.% CNF	267.56	+4.21%
0.25 wt.% CNF (water seal)	279.31	
0.5 wt.% CNF	263.50	+3.92%
0.5 wt.% CNF (water seal)	274.24	

**Table 4:** Results of restrained ring tests.

Mixture	Crack Onset t (d)	Strain Rate ((m/m)/Days 1/2)	Relative Change (Compared to Neat Paste)
W/C = 0.3, Neat paste	1.12	239.74	0
W/C = 0.3, 0.05 wt.% CNF	2.06	193.18	−19.4%
W/C = 0.3, 0.1 wt.% CNF	12.86	44.77	−81.3%

**Table 5:** A summary of potential working mechanisms of cation-modified CNF.

Hypotheses	Key Evidences	Confidence Level
Seeding effect	Insignificant increase in compressive strength at early age (−) No silica element (−) No obvious hydration product surrounding carbon element in BSE image (−)	Unlikely
Mechanical reinforcement	Lower flexural strength (−) Insignificant increase in microhardness (−)	Unlikely
Crack bridging effect	Sufficient fiber length (+) Lower flexural strength (−)	Unlikely
Internal curing effect	Significantly increased crack onset time in sealed ring tests (+) Increased hydration rate during the first 10 h (+) Significantly higher 28-day compressive strength (+)	Very likely
“Short circuit diffusion”	“Shell” region in the BSE image (+) Higher hydration heat release in water sealed samples (+)	Likely

## Data Availability

The original contributions presented in this study are included in the article. Further inquiries can be directed to the corresponding author.
